# Dexmedetomidine and midazolam usage and association with BSRI scores in preterm infants with chronic respiratory failure

**DOI:** 10.1038/s41372-026-02567-3

**Published:** 2026-02-23

**Authors:** John Mascari, Spencer Millen, Tanvi Batish, Karol Suarez, Susan Slattery, Megan Lagoski, Natalia Henner, Janice Raucci, Nicolas Porta, Karna Murthy

**Affiliations:** 1 Ann & Robert H. Lurie Children’s Hospital of Chicago; Feinberg School of Medicine, Northwestern University, Chicago, IL USA; 2 Ann & Robert H. Lurie Children’s Hospital of Chicago, Chicago, IL USA

**Keywords:** Outcomes research, Respiratory tract diseases, Paediatrics

## Abstract

**Objective:**

To quantify midazolam (MID) and dexmedetomidine (DEX) exposure in preterm infants with chronic respiratory failure (CRF) due to severe bronchopulmonary dysplasia (sBPD) and assess associations with Behavioral Signs of Respiratory Instability (BSRI) scores.

**Study design:**

Infants born <32 weeks’ gestation with sBPD requiring tracheostomy and chronic ventilation were included. Daily MID and DEX exposure was quantified from NICU admission until discharge, death, or transfer. Multivariable mixed-effects models evaluated associations between medication exposure and weekly BSRI scores, adjusting for selected confounders.

**Results:**

Among 40 infants (median NICU stay: 202 days), median exposures were 4.4 weeks for DEX and 2 weeks for MID, with peak doses surrounding tracheostomy. Medication exposure was not independently associated with BSRI scores. Higher respiratory severity scores correlated with increased medication doses.

**Conclusion:**

Neurosedative medication dosing correlated with disease severity. These findings highlight the need to optimize dosing strategies and neurodevelopmental outcomes in this vulnerable population.

## Introduction

Bronchopulmonary dysplasia (BPD) is the most common cause of morbidity and mortality in premature infants, and confers risks of death, cardiopulmonary morbidities, and adverse neurodevelopmental outcomes in early childhood [1]. Infant survivors of the most severe phenotype of chronic respiratory failure (CRF) can receive chronic mechanical ventilatory support for months to years after hospitalization in the neonatal intensive care unit (NICU) [2–4]. To manage these extended periods of intubation and invasive ventilation, neurosedative medications are frequently administered to provide comfort and tolerance of respiratory support [5, 6]. However, the extent of exposure to these medications remains insufficiently characterized [7].

While several studies have quantified the use of neurosedative medications due to concerns with side effects and potential impacts on neurodevelopment, data on cumulative dosages during NICU stays are limited [8, 9]. These medications are administered during a critical window of brain development, raising concerns about their potential to hinder optimal neurodevelopment in an already at-risk population [8, 10]. Understanding cumulative dosages can inform strategies to minimize the use of these medications, particularly as they are not directly linked to improved pulmonary outcomes in infants with BPD and CRF [11].

Correspondingly, our main goal was to quantify the daily exposure of two commonly used medications in our NICU, midazolam (MID) and dexmedetomidine (DEX). To quantify any associations between medication exposure and an infant’s inpatient developmental progress, weekly Behavioral Signs of Respiratory Instability (BSRI) scores, a previously described marker of an infant’s ability to participate in inpatient developmental therapy, were analyzed [12]. We hypothesized that the use of these medications would be both prolonged and significant during the NICU hospitalization and would be associated with markers of increased illness severity in infants with CRF and decreased BSRI scores.

## Methods

### Cohort selection

We performed a retrospective cohort study at our urban, academic regional, level IV neonatal intensive care unit (NICU). We included subjects who completed their NICU admission between January 2021 and October 2023 and were diagnosed with sBPD (defined as infants receiving > 2 L/min via nasal cannula or positive pressure respiratory support at 36 weeks’ post-menstrual age (PMA). Since our primary interest was to focus on the most severely affected infants with CRF, we further restricted the cohort to those who received a tracheostomy and chronic mechanical ventilation during their NICU hospitalization. We excluded infants born > 32 weeks’ gestation.

We considered the benzodiazepine (BZ) medication, midazolam (MID), and the alpha-adrenergic receptor agonist (α2-RA) medication, dexmedetomidine (DEX). Other benzodiazepines were not considered since we generally use them to facilitate weaning infants from continuous infusions. Opiate medications were not considered. While infants with CRF in our practice universally receive multiple medications, including opiates for analgesia, detailed data on these exposures were not collected, as the study focused specifically on DEX and MID. In each subject, medication exposure was restricted to the admission in which the tracheostomy procedure occurred and doses were recorded from the day of NICU admission until discharge, death, or transfer to another unit. Medication use and dosage were extracted manually from the electronic health record for each day an eligible infant was hospitalized.

We reviewed continuous infusions, pump boluses, scheduled doses, and as needed (PRN) doses of the selected medications. A flowsheet containing every rate change of continuous infusions was used to calculate the total daily dose of continuous medications based on their concentrations. For MID, we included all routes of administration including intravenous, enteral, and intranasal. The total absolute amount of each medication was recorded daily and converted to per-kilogram dosages based on the dosing weight of the patient on that day. In our practice, dosing weights are updated twice weekly, though if patients are significantly hypervolemic, these weights are subjectively modified by the clinical team. Our main metric for medication exposure is daily cumulative dose as a function of post-menstrual age (PMA).

### Outcome

The primary outcome was weekly Behavioral Signs of Respiratory Instability (BSRI) scores, a validated scoring tool to monitor developmental progress and respiratory stability during rehabilitation therapies [12]. This tool comprises of five domains (interaction, midline, movement pattern, tachypnea, and work of breathing) that reflect both behavioral and respiratory status. In our NICU, a core group of less than 5 primary therapists (PT/OT/SLP) record BSRI scores at least weekly once a patient reaches 40 weeks’ PMA. Prior studies have suggested that these scores can be reflective of affected infants’ responses to medical interventions, and given the known sets of multi-modal therapies administered to these infants, BSRI assessments may serve as a candidate marker to responses to these treatments in the NICU setting. Of note, the original BSRI validation study excluded infants with tracheostomies [12]. BSRI assessments were not done during periods when patients received pharmacologic muscle relaxation.

### Data analysis

Cumulative doses of DEX and MID were summarized as weekly exposures to align with the weekly assessments of the BSRI, and respiratory severity score (RSS) defined as mean airway pressure (MAP) × fraction of inspired oxygen (FiO2). A multivariable model was created to examine the correlation between individual patient’s exposure to DEX or MID and markers of disease severity. As patients varied widely in their dates of admission and discharge, the week (PMA) of highest exposure was chosen as the standardized comparison point for assessing associations between medication exposure and disease severity. Markers and correlates of disease severity chosen included RSS, post-menstrual age (PMA), gestational age at birth (GA), and small for gestational age (SGA) < 10th percentile [13]. These variables were selected based on their clinical relevance and potential relationship and impact on both the medication usage and patient outcomes.

The main outcome, BSRI score, was described as a function of PMA for included infants. Each infant was admitted at varied time points relative to birth given the usual practices toward our regional, referral-based NICU. Cumulative daily medication exposure by DEX and MID were described for each infant, and then, these data were collapsed by PMA, aligned with BSRI scores to observe/describe correlations with BSRI scores for ages > 40 weeks’ PMA.

Then, a multivariable mixed-effects model was employed to assess the association between medication exposure and BSRI scores. Random intercepts for each patient were included to account for intra-subject correlation. To account for suspected temporal autocorrelation of BSRI scores, we incorporated an autoregressive component (i.e., the BSRI score from the previous week) as a predictor in the model. Covariables include RSS, SGA, and PMA.

All statistical analyses were performed using R Version 2024.09.0 + 375. The mixed-effects model was fitted using the lme4 package. Model assumptions were checked by examining residual plots and assessing the normality of residuals. This study was exempted by the Institutional Review Board of the Ann & Robert H. Lurie Children’s Hospital of Chicago (IRB# 2023-6109).

## Results

Seventy-five patients received tracheostomies and completed a NICU admission between January 2021 and October 2023. Of these patients, 40 infants born <32 weeks’ gestation met inclusion criteria (Table 1). The median length of stay of the NICU admission in which the patient received their tracheostomy was 202 days prior to discharge home, death, or transfer to a different unit. Most (38/40) survived to discharge or inter-facility transfer. All patients were born at other centers and referred at a median PMA of 38.6 weeks (25–75th percentile = 31.8, 44.9) to our hospital. The median age of tracheostomy was 48.4 (25–75th percentile = 46, 52.6) weeks’ PMA.

**Table 1:** Characteristics of study participants.

Characteristics	Value
Total patients, *n*	40
Male, *n* (%)	23 (58)
SGA, *n* (%)	11 (28)
Survivors, *n* (%)	38 (95)
Gestational age, weeks (IQR)	26 ^0/7^ (24 ^2/7^–27 ^6/7^)
Birthweight, grams (IQR)	715 (542–945)
Length of stay, days (IQR)	202 (126–274)
PMA at admission, weeks (IQR)	38 ^4/7^ (31 ^6/7^–44 ^6/7^)

*N* number, *SGA* Small for gestational age (birthweight <10th percentile), *PMA* Post-menstrual age, *IQR* Interquartile Range, 25–75th percentile.

**Fig. 1:**
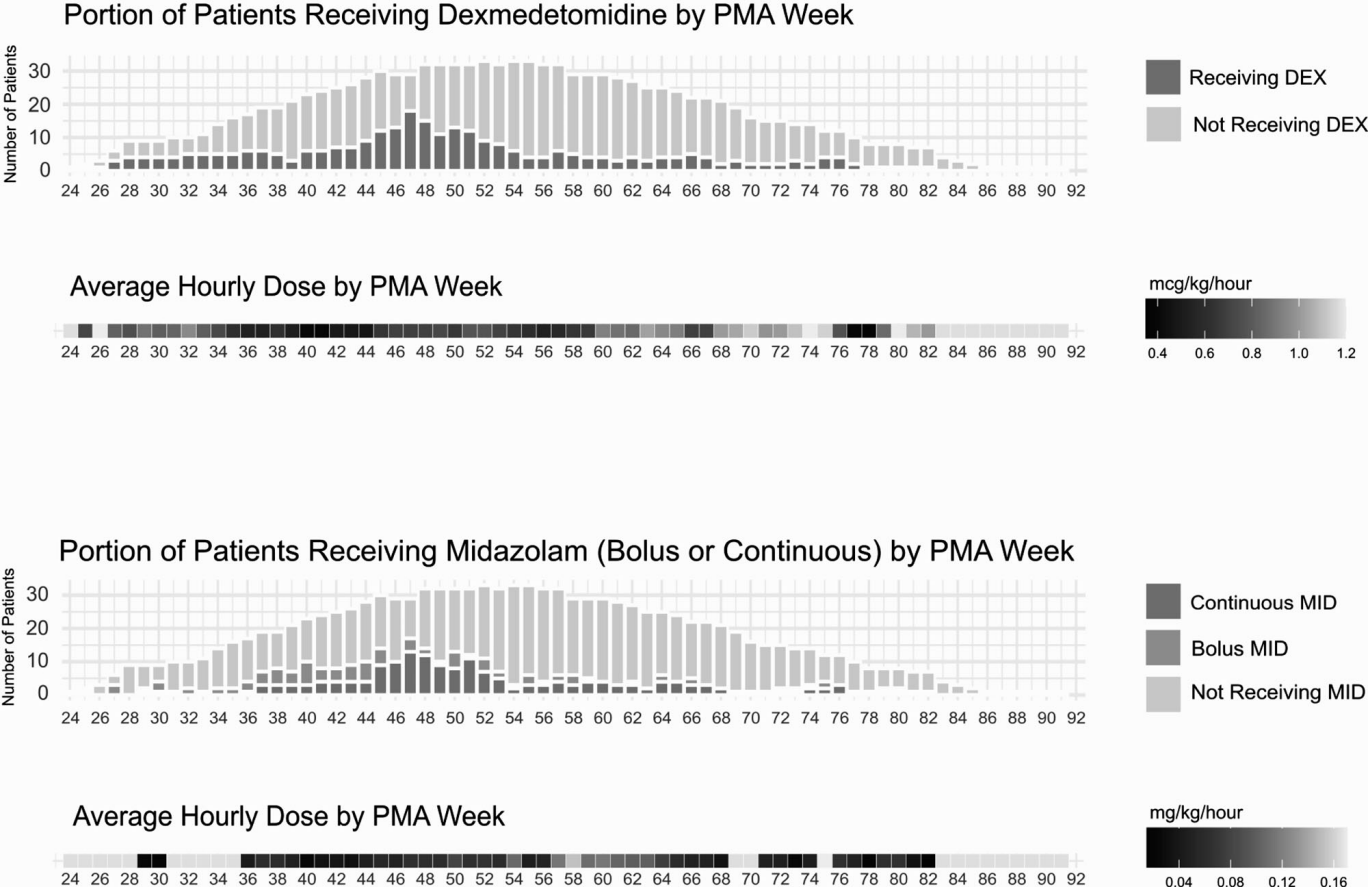
Heatmap of dexmedetomidine and midazolam usage by patient count and average dosage across PMA weeks. The number of patients receiving dexmedetomidine (top) and midazolam (bottom) for each PMA week is shown on the top with the corresponding average dosage of each PMA week shown on the bottom.

DEX and MID usage is shown in Fig. 1. These infants received DEX (*n* = 37) and MID (*n* = 38) for a median of 4.4 and 2 weeks, respectively. Out of the 40 infants, 33 (82.5%) received DEX and MID simultaneously for a median of 13.0 days (IQR 7.0–28.0). Average continuous infusion doses were 0.70 mcg/kg/hr for DEX and 0.06 mg/kg/hr for MID. For PRN dosing of MID, the average cumulative daily dose was 0.14 mg/kg. For both medications, doses peaked around the time of tracheostomy placement and then decreased with an average maximum dose at 48.4 and 50.5 weeks’ PMA for DEX and MID, respectively. These figures demonstrate the median starting and ending PMA when exposures to these medications were incurred.

**Fig. 2:**
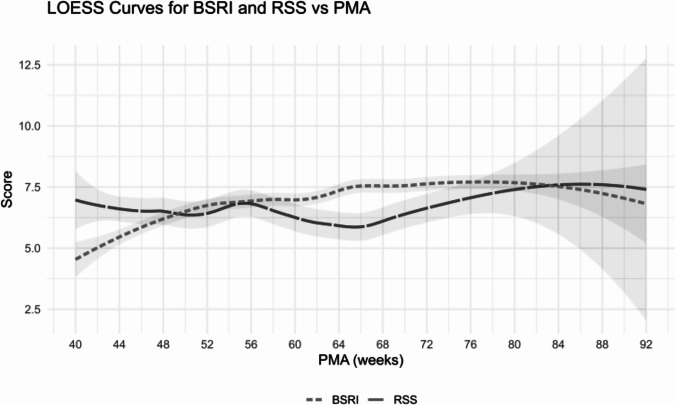
BSRI and RSS vs PMA. LOESS (locally estimated scatterplot smoothing) was applied to BSRI and RSS scores across 40–92 weeks PMA, with predicted values and 95% confidence intervals displayed as smoothed curves.

The main outcome of this analysis was infants’ BSRI score (Table 2) and its association with medication dosages. Autoregression was used to account for autocorrelation of BSRI scores from the preceding week. We found no relationship between medication dosages of either DEX or MID and BSRI scores. First, the impact of the daily dose of each medication at a given week on BSRI scores was determined. Next, the relationship between the cumulative dosage a patient had received up to the previous week and BSRI scores was compared. After accounting for historical BSRI, PMA, SGA < 10th percentile, and the weekly measured RSS, neither the cumulative nor current doses of either medication were associated with BSRI scores. BSRI and RSS scores as a function of PMA are shown in Fig. 2.

**Table 2:** Factors influencing weekly BSRI scores based on multivariable mixed effects model using autoregression.

Factor	β	95% CI		*P*
Historical BSRI	0.87	0.84	0.90	<0.001
RSS	−0.01	−0.03	0.00	0.056
Current dexmedetomidine dose	0.00	−0.001	0.001	0.596
Current midazolam dose	0.00	−0.01	0.01	0.993
SGA (*n*)	−0.07	−0.18	0.03	0.172
PMA (weeks)	0.01	0.00	0.01	0.001

Random intercepts for each patient were included to account for intra-subject correlation. To account for autocorrelation of BSRI scores, autoregression was incorporated by using the BSRI score of the previous week as a predictor starting at 40 weeks PMA.

*BSRI* Behavioral Signs of Respiratory Instability, *RSS* Respiratory Severity Score, *SGA* small for gestational age, *n* number, *PMA* post-menstrual age.

In secondary analyses, the association between dosage of DEX or MID and markers of disease severity are shown in Tables 3 and 4, respectively. We found no correlation between DEX dosage and GA, SGA, or PMA, but a significant association with RSS (10.8 mcg/kg/week increase per RSS unit). MID showed a similar pattern, with each RSS unit increase linked to a 1.07 mg/kg/week dosage rise.

**Table 3:** Factors associated with PMA week of highest total dexmedetomidine exposure based on linear regression.

Factor	β	95% CI		*P*
RSS	10.8	6.70	14.9	<0.001
SGA (*n*)	−33.9	−79.2	11.3	0.151
Gestational age (weeks)	−7.84	−17.1	1.47	0.108
PMA (weeks)	1.06	−0.99	3.11	0.319

The PMA week of highest exposure was chosen as the standardized comparison point for assessing associations between medication exposure and disease severity. The beta coefficient is expressed in mcg/kg/week of dexmedetomidine.

*RSS* Respiratory Severity Score, *SGA* small for gestational age, *n* number, *PMA* post-menstrual age.

**Table 4:** Factors associated with PMA week of highest total midazolam exposure based on linear regression.

Factor	β	95% CI		*P*
RSS	1.07	0.55	1.59	<0.001
SGA (*n*)	2.76	−2.24	7.76	0.286
Gestational age (weeks)	−0.50	−1.57	0.57	0.369
PMA (weeks)	0.28	−0.01	0.56	0.063

The PMA week of highest exposure was chosen as the standardized comparison point for assessing associations between medication exposure and disease severity. The beta coefficient is expressed in mg/kg/week of midazolam.

*RSS* Respiratory Severity Score, *SGA* small for gestational age, *n* number, *PMA* post-menstrual age.

## Discussion

Preterm infants with CRF associated with severe BPD are at high risk for adverse outcomes in early childhood including abnormal pulmonary function, as well as cardiopulmonary and neurodevelopmental morbidities [14, 15]. Many studies have described associations between BPD and adverse neurodevelopment, however, few have focused on either the most severely affected infants with BPD or the dosages of neurosedatives administered during the NICU hospitalization despite their likely contribution. In our study, we detailed the daily dosage of DEX and MID in a local cohort of preterm infants with CRF during a critical time of neonatal brain development. We did not observe an independent association between medication exposure/dosage and short-term BSRI scores; instead, our results suggest that the severity of respiratory disease (indexed by RSS) was a likely contributor to dosage escalations for these infants.

This study offers a novel, comprehensive view of our patients’ exposures to DEX and MID in the context of sBPD and CRF. We included continuous infusions, boluses, scheduled doses, and PRN doses, with total dosages normalized to each patient’s most recent dosing weight, allowing for meaningful comparisons over their hospitalizations. The majority of patients received both medications for prolonged periods at relatively high dosages during an important period of brain development that includes the formation of synaptic and neuronal connections and the expansion of structures like the thalamus, the cortex, and the cerebellum [16].

Our regression model accounted for both static factors and dynamic weekly clinical changes, such as RSS, BSRI, and cumulative dosage. Although no association was observed between neurosedative exposure and BSRI scores, it is important to note that the BSRI was not designed to detect clinically significant medication side effects. Additionally, the five domains of BSRI scores include both developmental as well as respiratory components which could both be independently influenced by medication dosage. Total daily dosage is a crucial metric for understanding neurosedative use, yet it has not been extensively reported in this population, making it challenging to draw conclusions about practice variations or the generalizability of our findings. It remains possible that higher dosages than those administered in our unit could be associated with changes in BSRI scores.

Various studies have investigated the long-term outcomes of exposure to neurosedative medications. The EPIPAGE study demonstrated that moderate to severe disability at 5 years of age was associated with prolonged sedation exposure in very preterm infants; however, after adjusting for gestational age this association was not observed [17]. Another study of 936 infants showed that prolonged exposure to morphine, fentanyl, MID, or lorazepam was associated with lower Bayley Scales of Infant and Toddler Development III scores at 2 years of age compared with infants without exposure [8]. In the same study, use of benzodiazepines for more than 7 days was associated with lower cognitive, motor, and language scores at 2 years of age [8]. Preclinical studies have also shown neuroapoptosis as well as long-term functional deficits and atypical behavioral patterns associated with benzodiazepines [18]. In the context of these studies, our results suggest that MID dosage appears to have less, or potentially no observed influence on outcomes compared to the severity of CRF. More inquiry is required to disentangle these effects, particularly as dosages are potentially modifiable once CRF is established in these affected infants.

Perhaps because of its recent increase in use, there are no long-term studies that assess the developmental outcomes associated with DEX exposure during the neonatal period or early infancy [19]. Animal studies have yielded mixed results: one study in rat pups found that a single dose of DEX on postnatal day seven did not impair hippocampal synaptic plasticity at nine weeks of age [20], while another study suggested that DEX inhibited neuronal apoptosis and suppressed cytokine-mediated brain injury [21]. Our results suggest that DEX exposure is not related to short-term development, but clearly this retrospective first analysis would greatly benefit from prospective observation through early childhood.

This study has several limitations including its retrospective design and the modest sample size. The cohort’s multiple comorbidities and higher likelihood of receiving prolonged, high-dose neurosedative medications limit the generalizability of our findings to the broader NICU population. We did not specifically address the effects of concurrent versus non-concurrent medication use or the interactions with other drugs (e.g., opioids, barbituates, ketamine, gabapentin), due to their inconsistent use and data collection challenges. It is possible that the use of MID interfered with or modified infants’ controls of breathing, leading to clinical assessments that may alter ventilatory supports and perhaps drug dosing/exposures. Additionally, unmeasured or unknown factors such as feeding tolerance may have modified or confounded the observed associations. Long-term developmental outcomes were not assessed, and while BSRI scores are reproducible, their clinical assessment and measurement includes subjectively assessed components. As previously mentioned, BSRI has not been validated in patients with tracheostomies, but we believe the scale’s focus on behavioral and respiratory instability remains applicable to our cohort given the shared pathophysiology of BPD and chronic respiratory support.

In conclusion, our patients with severe BPD and CRF receive high doses of DEX and MID for prolonged periods of time during critical periods of neurodevelopment. Higher RSS was associated with higher dosage, but higher dosage was not associated with changes in weekly BSRI. This finding raises the question of what the primary purpose of neurosedative medication is and how dosages should be titrated. Without publicly available data on neurosedative usage, such as those presented in this study, center-to-center variation cannot be studied making practice guidelines difficult to standardize. Neurosedation management is a growing concern that is multifactorial, complex, and deserving of further studies.

## Data Availability

The datasets generated and analyzed during the current study are not publicly available due to technical limitations, but are available from the corresponding author on reasonable request.
